# Medical and Physical Intelligence

**Published:** 1825-05

**Authors:** 


					MEDICAL AND PHYSICAL INTELLIGENCE.
PHYSIOLOGY.
1. On the Sensibity of the Nerves of the Senses.?M. Magendie,
at a recent meeting of the Institute, communicated verbally some obser-
vations on this subject. He is of opinion that the sensibility of the
nerves of the senses is entirely relative to the kind of impression which
they are destined to communicate; that the retina, for example, is only
sensible to light, and that it may be pressed, pricked, or torn, in ani-
mals, without exciting pain. A short time ago, in operating for cataract
on a woman, M. Magendie accidentally touched the retina, without his
patient manifesting any sensation. Having afterwards touched the re-
tina of both eyes, he ascertained, in the most satisfactory manner, that
this part is as insensible in man as in other animals. The woman did
well.?prf Bulletin des Sciences Medicates, Mars.)
2. On the Galvanic Phenomena which accompany the Acupunctura-
tion.?M. Pelletan has ascertained, by means of the galvanom&tre
of M. Bicquerel, that perceptible quantities of this fluid are always
disengaged by a needle plunged into a part of the human body affected
with pain. The quantity is extremely small; perhaps, says M. Pelletan,
not equalling the hundredth part of what is obtained from a single plate
of a common voltaic pile. Nevertheless its effects maybe rendered
sensible; for which purpose, it is sufficient to make the needle inserted
into the affected part communicate with the mouth, by means of a me-
tallic wire. M. Pelletan thinks these galvanic phenomena unconnected
with the curative effects of the operation; which opinion is founded upori
the circumstance of the relief obtained by the patient being in no case
in proportion to the quantity of the fluid disengaged; and that very
marked effects result from the acupuncturation, even with a needle ter-
minated by a non-conductor. No perceptible difference presents itself
with regard to the degree of relief between the use of needles ending in
non-conductors, and those so arranged as to allow the electricity to pass
off. It is asserted by M. Pouillet, that no galvanic phenomena
present themselves when needles made of platinum or gold are enir
ployed; from which he infers that, in the cases alluded to, the effects
were the result of the oxidation of the metal. M. Pelletan has seen
incontestible success follow the use of the acupuncturation, particularly
in rheumatic affections. ? (Ibid. February.)
Medical and Physical Intelligence, 435
PATHOLOGY.
3. Rupture of the Spinal Marrow.?M. Andral has related, ou
the authority of M. Velpeau, a case of disorganisation of the spinal
marrow, which gave rise to no symptoms by which the existence of so
severe an affection could have been suspected. The singularity of this
fact, and the deductions drawn from it by the author, gave rise to a dis-
cussion at the Royal Academy of Medicine, in the course of which
various members related instances of the complete destruction of organs
important to life, in which no suspicion was entertained of the nature
of the disease, until it was ascertained by examination after death.
Among others, the case was related of an individual long affected with
hemiplegia, in whom the most minute dissection discovered no vesjiige
of morbid alteration : the brain and spinal marrow, the circulating sys-
tem and lungs, the digestive organs and the kidneys, were successively
and ineffectually examined.?(Revue Medicale, Mars.)
4. Unusual Appearance of the Blood.?M.Velpeau relates the
case of a man, robust and of good constitution, who had never experi-
enced any severe illness during his life: he had been much given to
venereal indigencies, but had not suffered from syphilis. At fifty-seven
years of age he fell upon his right side, and was not restored to healtU
for sixty-five days. Soon after he attained his sixtieth year, he observed
that the left side of his abdomen increased in size, and he f<jlt in that
situation a tumor, which did not, however, become painful until the end
of December last. From that time his health declined, without any
particular malady ;?he continued to eat and drink, and was not
confined to his bed. On the 16th of February, he felt a sensation of
heat rising to his head; soon after, his face assumed a bluish cast; he
complained of head-ache and deafness, but did not lose his recollection.
After some remedies had been ineffectually tried, he died on the lpth, at
three o'clock in the morning. On opening the body, all the internal
membranes appeared very highly coloured, but without thickeniug or
alteration of texture. The colour was derived from vessels gorged with
a thick blood, the colour of the lees of red wine. The same coloured
fluid filled the vena cava, the aorta, the auricles, and the heart. The
blood was not coagulated, neither could it be called fluid ; it was nearly
the consistence of bouillie ; it was of a reddish.black colour, analogous
to that of the lees of pure red wine, or to that of the matter of abscesses
which are sometimes formed in the liver. This was the case over the
whole.body; in no part did the blood present its natural character.
The spleen weighed ten pounds; the liver was double its usual bulk ;
but neither of these viscera were disorganised, nor was there any altera-
tion of texture to be found in any part, excepting some ulcerations in
the descending aorta, and some of its priucipal branches; the veins
were healthy. It may be remarked that the change in the appearance
of the blood was such as to induce a question whether the vessels were
not filled with purulent matter.?(Archives generates, Mars.)
5. Strangulation of aportionof the small Intestines.?M. Esquirol
relates a remarkable case of strangulation of a portion of the small
436 Medical and Physical Intelligence.
intestines. An accidental bridle united the broad ligament of the uterus
on the right side with the rectum on the same side. A portion of in-
testine had passed, in the first place, between this bridle and the sacrum,
afterwards through the same band and the anterior parietes of the
abdomen, and had lastly entangled itself between the sacrum and the
bridle, so as to form a kind of running knot. The woman who had
been the subject of this strangulation died, at the end of a few days, of
the symptoms which this accident usually produces.?(Ibid.)
THERAPEUTICS.
? 6. On the Use oj the Oil o/Croton in Inflammation of the Bowels.?
Professor Morichini, of Rome, wishing to ascertain whether the use
of this remedy might be permitted in inflammatory affections of the
bowels, prescribed it in two cases of gastro-enteritis. They, were both
females. One drop of the oil was mixed with an ounce of simple syrup,
and administered in two doses, with an interval of two hours between
cach. The first patient did not experience any sensation of heat in the
fauces. Two hours after, the bowels were moved, and during the night
thirteen stools were procured, which produced much prostration of
strength, but no pain comparable to that which she had suffered. The
other patient, aged seventy-five, when seen by the Professor, had fever,
with acute pain and tension in the abdomen, hard pulse, flushed coun-
tenance, anxiety, nausea, and constipated bowels, for seven days;
although many glysters, with oil and common salt, had been thrown up.
At three o'clock she was bled to the amount of twelve ounces; had her
abdtonien fomented, and an oily embrocation used, besides glysters of
decoction of marshmallow and oil. At eight o'clock in the evening, one
drop of the oil of croton, in an ounce of syrup of marshmallows, was
ordered to be given in two doses, with an interval of two hours between
each, provided the first produced no effect. The first dose, however,
procured seven evacuations, which followed each other rapidly. The
woman slept afterwards; and, though some fever remained the next
day, it was only necessary to continue the fomentation, with demulcent
drinks, to complete the cure.
From the result of these cases, the Professor is inclined to think that,
although it would not be proper to administer this medicine in the height
of an inflammatory attack, yet when, by means of bleeding, copious
warm dilution, and other means, abdominal inflammation is in some
measure allayed, the oil of croton may be administered, without any
apprehension of reviving the phlogistic state,? or rather, indeed, with a
certainty of overcoming it altogether.?(Annali Universali, Dec.)
7. New Species of Cinchona.? Mr. Dupau has read, before the
Royal Academy of Medicine, an account of a new species of cinchona,
named bicolor, and resembling the cascarilla. Tiiis bark was sent to
him by M. Br ERA, who has experienced the good effects of it in cases
where the common bark had failed. Its action appeared to be more
energetic, but, by analysis, it was found not to contain quina. ,The
absence of this principle leads to the belief that it is not really belonging
to the genus cinchona.?(Archives generates, Mars.)
Medical and Physical Intelligence. 437
8. Disappearance of the Mammce, caused by the Use of Iodine.?
M. Hufeland relates among other examples of this misfortune, that
of a girl, twenty years of age, naturally of good constitution, who used
for about six months the tincture of iodine, for the removal of a goitre.
It produced the desired effect; but she perceived that the mammae were
depressed, and had diminished in size. Notwithstanding the disconti-
nuance of the iodine, this wasting progressively advanced, so thai, at the
end of two years, no vestige of the mammary gland remained. The
same author has known two other analogous instances.?(Journal der
Prakt. Heilkunde.)
SURGERY.
9. Ophthalmia cured by Acupuncturution.?M. Cloouet lately
presented to the Royal Academy of Medicine the case ot'a young woman,
who had been affecled, for a very long period, with chronic ophthalmia
of the left eye, with puriform discharge from the eyelids. The eye was
constantly closed, and the affection complicated with acute pain in the
orbit and in the head. A variety of means had been employed without
success, when M. Cloqnet had recourse to acupuncturation. He inserted
two needles in the temporal region of the same side : they caused but
little pain, and at the end of a few days the iullainmation had sensibly
diminished ; but it was especially the pain in the orbit and head, which
disappeared almost instantly. In a few days they again returned. He
then introduced another needle into the middle of the forehead, and left
it there. The eye speedily improved, remaining open; the pain disap-
peared. There existed at the same time an eruption on this side of the
face, which was almost entirely removed.
M. Husson communicated to the same Body the case of a very stout
man, who was admitted at the Hotel Dieu for an ophthalmia of the
right eye, which was entirely closed and extremely painful. This in-
flammation, which had resisted during eight days all the most active
remedies, disappeared very rapidly on the introduction of two needles
into the thick part of the eyebrow on the right side.
M. Nacquart afterwards communicated the case of a young lady,
affected with a painful ophthalmia of long standing, in which the acu-
puncturation had been practised twelve days before, without any benefit
resulting. It appears, however, that, from the modification in the ope-
ration employed, it rather resembled the introduction of a seton, than
the acupuncturation properly so called.?(Revue Med. Mars.)
MISCELLANEOUS.
10. Unpublished MS. of Morgani.?Dr. Frank has lately ad-
dressed a letter from Parma, to M. Des Genetts, of Paris, in which
he announces that he has found, in the library of the Grand Duke,
twelve sets of manuscripts of Morgani, relative to anatomical re-
searches and to consultations. They are, for the most part, written in
ihehandof this distinguished anatomist,and belonged to Dr. Ricciardi.
Eleven sets are devoted to anatomy, and the twelfth to consultations.
There is reason to hope that they will soon be given to the public.
438
A Case of Irritative Fever, arising from a Scratch received in a
Morbid Dissection ; with Remarks on the Nature and Treatment
of similar Cases. By Anthony Todd Thomson, m.d., f.l.s.
&c. &c. [From the London Medical Repository, April 1.]
Whatever may be the cause, the fact is well ascertained, that more medi-
cal practitioners have suffered from accidents depending upon morbid
dissections within these last ten years, than for half a century before the
commencement of that period ; yet, it is a remarkable circumstance, that,
amongst the numbers who have recovered from these accidents, no indivi-
dual has published his case for the benefit of the profession. Having lately
almost lost my life from an accident of the above-mentioned kind, I shall
endeavour to supply the desideratum which I have noticed, by giving you
a detailed account of my own case, with a few remarks on the nature
and treatment of similar cases.
Early 111 the month of November last (1824), I was requested to see a
lady who had been labouring, for ten days, under a severe inflammatory
affection. I found her in a slate of extreme danger; and, indeed, so
hopeless was the case, that I hesitated to use the lancet, on which alone,
however, any prospect of benefit depended; for, although she had been
previously bled, both topically with leeches and at the arm, yet, the ex-
citement was such, that nothing but further depletion promised even a
shadow of success. She was, accordingly, bled to the amount of twenty
ounces and purged freely ; but she died on the following day. I may men-
tion, that the inflammation under which she laboured, when my advice was
demanded, was pleuritic: but, as her friends informed me that her head
had been previously the seat of the disease, and it was only when the
pain left the head that the pleura became alfected, I concluded that the
disease was rheumatism, and that her dissolution was to be attributed to
the metastasis which, sometimes, occurs in the acute form of that com-
plaint. It may be proper to mention, also, that the arm in which she had
been bled, previously to my seeing her, was painful, much swelled, and
displayed all the symptoms of inflammation of the vein ; to relieve which,
it had been poulticed and kept cool with an evaporating lotion. She had
been delirious during the night before the day in which I saw her; but, at
the time of my visit, she was perfectly collected, and continued so until
the moment of her death, which was unattended by any struggle or convul-
sion. On the second morning, at half-past seven o'clock, thirty-two hours
after her death, I opened the body.
1 he stomach, although it was greatly distended with flatus, yet was
perfectly healthy in appearance; as was the liver, the pancreas, the spleen,
and, with the exception of one of the ovaria, every abdominal viscus.
The uterus was unimpregnated, and the os tincae completely pervious; the
right ovarium was rather larger than usual; and a hydatid, the size of a
pea, was appended to it by a kind of a ligament. I particularly notice the
unimpregnated state of the uterus, because an opinion prevails, that much
of the danger arising from the wounds received in morbid dissections de-
pends on something connected with the puerperal state. On opening the
thorax, its left cavity was found to contain about a quart of bloody serum.
The pleura costalis, from the third to the seventh rib, and from the spine
to about the breadth of the hand distant from the sternum, bore evident
marks of violent inflammatory action having recently existed, as did also
the pleura pulmomum, corresponding to the above-mentioned space; both
were covered with a reticulated web of coagulable lymph, but in no place
did ihcy adhere. The greater part of the principal lobe of the left division
of the lungs was turgid with blood. There were no other appearances in-
dicative of disease in any part of the bodv.
6
Dr. Thomson's Case of Irritative Ftver. 439
No accident occurred, either to myself or the young gentleman who as-
sisted me, in the dissection; but, in sewing up the body, having, impro-
vidently, furnished ourselves with curved needles only, I received a slight
scratch on the first joint of the fore-finger of the right hand, owing to the
needle turning suddenly round whilst I was forcing it through the integu-
ments of the body. The wound was so slight that I paid no attention to it
at the time it occurred ; nor was it sufficient to attract my notice until the
evening, twelve hours afterwards, when it excited a slight degree of pain
and appeared a little iullamed. The pain, however, increased towards bed-
time ; awoke rne after I had been asleep two hours only; kept me awake
the rest of the night, and was accompanied with very profuse perspiration.
In the morning the finger was considerably inflamed, and a small white spot
appeared in the centre of the scratch, from which, on opening it with the
point of a lancet, I squeezed a globule of pus. The finger was so much
relieved after this slight operation, that I regarded the wound unworthy of
farther notice; but, during my professional visits in the forenoon, I was at-
tacked with rigors; my strength gradually failed, and my system became
evidently under the influence of incipient fever. I, nevertheless, continued
my visits, although I was, at length, scarcely able to ascend a stair; and,
on returning home at two o'clock in the afternoon, I fainted as I was giving
my orders to my assistant; but boing laid upon a sofa I soon revived, and
became sensible of the nature of my alarming condition.
Although my mind was weakened by the state of my body, I was suffi-
ciently collected to reflect, that as extreme prostration of strength was the
most marked symptom of my attack, the best method of meeting it was to
attempt to rouse the nervous energy, and, at the same time, to clear out the
bowels: I therefore ordered for myself a mixture containing camphor, am-
monia, and wine of colchicum; and had taken two doses of it before my
friend Dr. Granville, who had been sent for on the occasion, arrived. I
could not convey a more accurate idea of my sensations to the doctor, than
by comparing them to those which are said to result from the bite of a
Cobra di capella, or other venomous serpent; or to those which I had once
beard described by a person who had taken an overdose of prussic acid.
.The debility, besides being excessive, was accompanied by such a feeling
as I conceive must attend the approach of death at the close of a disease of
debility. The respiration was laborious, and accompanied by an acute
pain under the xiphoid cartilage, extending to a short distance along the *
sternum ; while the pulse was quick, vacillating, and struggling, with occa-
sional hard throbs, "which," to use Dr. Granville's words, " would have
authorised bleeding in the hands of an inexperienced practitioner.1'
Dr. Granville concurred in the principle which had guided me in prescri-
bing for myself, but disapproved of the colchicum; and, instead of the
mixture which I was taking, ordered for me a bolus composed of three
grains camphor, and four grains cayenne pepper; and, as my extremities
were cold, the entire surface of the body pale, and the features shrunk and
cadaverous, he directed the feet to be bathed in hot water previous to my
being conveyed to bed. The prostration of strength, was, however, so
great, that this could be effected only whilst 1 remained in the recumbent
posture, in which position, also, my clothes were obliged to be taken off.
The wounded finger was poulticcd.
The bolus, which was repeated, and the pediluvium, produced that reaction
which was expected, and I obtained some sleep during the night. On the
following day the pulse was upwards of 130, but small; the skin hot and
dry : and there was some degree of delirium ; but these symptoms, as well
as the pain under the xiphoid cartilage, which was still felt, although in an
inferior degree, in the morning of this day, subsided as the bowels were
freely opened, by a calomel bolus and a brisk purgative, composed of a
NO. 315. 3 L
440 Dr? Thomson's Case of Irritative Fever.
scruple of jalap, and half a dram of supertartrate of potass, which Dr.
Granville had prescribed. The pain of the finger, which was now swelled,
stiff, and inflamed, was less severe than I anticipated; but it extended up
the arm, and was slightly felt in the axilla. An evaporating lotion was
substituted for the poultice, and three grains of James's powder were or-
dered this evening in combination with four of extract of henbane, instead
of the cayenne pepper. I slept better this night; and, by continuing the use
of the pills, and maintaining the full action of the bowels for the three fol-
lowing days, I was so much relieved as to be able to sit up for a few hours
and to take a little nourishment on the fifth day; and, on ihe sixth, I con-
sidered myself out of all danger. The pain of the finger which had hitherto
given me but little uneasiness, began now to be excruciating; and, not-
withstanding the use of warm fomentations and poultices, increased to a
degree which was almost insupportable. The seat of pain, however, was
not the wounded part, but nearly an inch above it, in the second phalanx
of the finger. My friend Mr. Brodie saw me late in the evening, in con-
junction with Dr. Granville; and as it was supposed that suppuration had
begun under the fascia, it was determined to lay it open by a free incision
down to the bone, which was accordingly done by Mr. Brodie; and the
hand was afterwards exposed to the influence of the steam of boiling water.
Scarcely more pus than was sufficient to cover the point of the lancet was
evacuated; but by continuing the steaming, at intervals, for several days,
the part suppurated freely, and my recovery was completed. At the end
of ten days I was again able to renew my professional avocations, although
some weeks elapsed before the debility in which I was left by the disease
was entirely overcome. I ought to mention that the middle finger of my
left hand, on which a small morsel of the cuticle close to the nail was
separated, forming what is vulgarly termed " a hang nail," became inflamed,
suppurated, and cast the nail.
The following corollaries are deduced from the facts connected with the
origin and progress of this case.
1st, It is evident that diseases of an inflammatory nature, affecting the
serous membranes, even when unconnected with the puerperal state, generate
a virus, which continues active for some time after the death of the indi-
vidual ; and is capable, when introduced into the living system by inocula-
tion, of exciting a dangerous degree of irritative fever.
2d, It is probable, however, that a certain predisposition of thtj liody oL
1 the dissector may be necessary for the production of this effect.
in my own case my health was not in tlie best state at the time of my
receiving the scratch, as I had been pveviously much harassed both in body
and mind, by the extent and nature of my professional duties.
3d, That the effect of the introduction of this virus into wounds, or chops,
or abrasions of the cuticle of the hands in dissection, is local inflammation
and the production of a similar virus in the part, which, being absorbed into
the system, diminishes so greatly the nervous energy, as nearly to destroy
the action of the heart; and, thence, to produce congestions in the vasculat
trunks highly detrimental to the powers of life, and which prove not un-
frequently fatal.
If these corollaries be correct, two questions of great importance present
themselves for consideration :?1st, In what manner, independent of care
in avoiding wounds, is the influence of this virus to be guarded against in
dissection? 2d, When inoculation has taken place, what plan of treatment
is likely to prove most beneficial in overcoming the disease which follows?
In considering the first query, it is obvious, that no description of gloves,
nor any coverings for the hands of a similar nature, can be employed by the
dissector; but I am of opinion, that the hands, if chopped or abraded,
might be protected by rubbing them over with oih It is a well-known
2
Dr. Thomson's Case of Irritative Fever. 441
fact, that the oil coolies at Tiirsis are rendered insusceptible of the contagion
of plague, owing to their bodies being constantly covered with oil, which can
be explained only on the principle of non-contact; and as oil is impermea-
ble by watery fluids, the probability is, that it would secure the chopped or
abraded hands of the anatomist, in morbid dissections on the same principle.
But it caunot secure him from being wounded either by the scalpel, the
needle, or the edges of fractured or of carious bones, or from the conse-
quent inoculation ; and, thence, the necessity of the second query.
The wounds which are received in morbid dissections are seldom deep,
and most frequently do not penetrate through the cutis vera. When this
is the case, perhaps the most certain mode of preventing the threatened
evil would be, instantly, to cut out the portion of wounded skin with a clean
scalped, and to encourage a flow of blood by bathing it with warm water.
Sometimes, however, scratches are received unconsciously, owing to the
attention of the dissector being deeply engaged in the investigation of his
subject, and the first notice he receives of thf; injury is from the loCal in-
flammation of the wounded part. It is then too late to have recourse to
excision: what mode of treatment, therefore, should be adopted ? Were I
to reason from my own case only, I might consider it sufficient to refer to the
treatment which it details; but the profession has been instructed, by expe-
rience, not to depend upon the result of single cases. If the view, how-
ever, which has been given of the nature of the attack be correct?if the
first effect of the absorption of the virus be to diminish the nervous energy,
and, by thus weakening the moving powers of the blood, to permit conges-
tions in the Irunks of the vascular system,?it is evident that, unless the
balance of the circulation be restored, the functions of life cannot be conti-
nued. Reaction may take place by the powers of the habit; or the con-
gestion may be removed by bleeding; or it may be overcome by reaction
induced by rousing the nervous system by artificial stimuli, as in the case
before us. If we trust to the powers of the habit, it is impossible to calculate
upon the consequences: the resulting fever may be sufficient to endanger
life, or organic mischief may occur, which would ultimately destroy it;
and it is unnecessary, therefore, to reason upon the probable effects of the
vis medicatrix in cases of this description. It only remains, therefore, to
estimate the comparative advantages of bleeding and of stimuli. The
opening a vein in the arm will undoubtedly relieve congestions in the vas-
cular trunks within the thorax and abdomen; but if these congestions have
arisen from the nervous energy being diminished, by the introduction into
the habit of a virus operating as a powerful sedative, it may be doubtful
whether, after they have been thus relieved, the balance of the circulation
will be maintained. If the moving power of the vascular system depends
on nervous energy, the mere unloading the great vascular trunks, in which
the blood has accumulated from the action of the heart being unequal to
propel it onwards, owing to a paralysis, as it were, of the nerves, is not
likely to restore that power; and, therefore, we must conclude, that the
lancet is less efficient than stimuli, which are calculated to rouse and
maintain the nervous energy. The result of the practice which has been
generally employed in these cases, might be brought forward to determine
the question ; but the records of it are too scanty to admit of a satisfactory
inference being drawn from it. In my own case, in which there was evi-
dently no injury either to a nerve or a vein, the beneficial effects of the
stimulating plan, in the first instance, followed by active purgation, was
decisive; and my friend Dr. Granville, to whose skill, judgment, and
kind attention, I attribute my recovery, has found it equally so iu several
cases which have since occurred, and come under his care. It is, at all
events, worthy of beiug tried by the test of experience.
442
METEOROLOGICAL JOURNAL,
From March 20 to April 19, 1825.
Mar
20
21
24
25
26
27
28
29
30
31
Apr.
1
2
3
4
5
6
7
8
9
10
11
12
13
14
15
16
17
18
19
Rain
gauge
30.48
30.43
30.17
30.02
29.93
29.70
29.88
29.93
29.90
29.82
29.88
30.10
30.30
30.31
30.21
30.10
30.11
30.18
30.20
30.23
30.08
30.12
30.00
29.88
29.87
29.95
30.00
29.97
30.10
30.06
30.06
30.48
30.31
30.06
30.02
29.77
29.73
29.95
29.86
29.90
29.80
30.00
30.23
30.33
30.26
30.10
30.10
30.15
30.20
30.21
30.15
30.04
30.06
29.93
29.96
30.03
29.99
30.04
29.96
30.12
30.01
30.06
UE
LUC'S
HYG.
9 A.M.
E
NE
E
NE
NE
SE
E
NE
NE
"SE
SE
ENE
NE
NNE
SW
W
E
E
E
ESE
E
NE
W
W
w
wsw
WNW
VV
NE
*NW
NE
10p.m.
E
ESE
E
NE
ENE
E
ESE
E
E
ESE
ESE
NE
ENE
E
NNW
ENE
E
E
E var.
NW
SW
w
NW
N
W
W
NW
ENE
ENE
WNW
ATMOSPHERIC
VARIATIONS.
9 A.M.
Fine
Cloud.
Fine
Foggy
Cloud.
Foggy
Fine
Fogey
Fine
Cloud.
Fine
Cloud.
Fine
2 P.M.
Fine
Cloud.
Fine
10p.m.
Fine
Cloud.
Fine
Foggy
Cloud.
Fine
Foggy
Cloud.
Fine
Cloud.
Fine
The quantity of rain fallen in March, was ll.lOOths of an inch.

				

